# Typhus Fever

**Published:** 1897-08-14

**Authors:** 


### TYPHUS FEVER.

The outbreak of typhus fever in the island of Inniskea, on the far west coast of Ireland, and the spread of the epidemic to the mainland, have drawn attention afresh to a disease which used to be one of the most fatal of London fevers, and now is almost forgotten. In 1895 only 15 cases of this disease were notified, five of them proving fatal, a number absolutely insignificant. Yet typhus fever is a most infectious malady, not in the way that plague or beri-beri or even typhoid, is infectious, but absolutely catching from man to man, as many a medical practitioner has found out to his cost; and it becomes one of the most interesting problems in epidemiology to trace the manner of infection by which so sparsely scattered a disease is, if we may say so, kept alive. Nothing could be more striking, as showing the preponderating influence of what one may call predisposing conditions in determining the spread or the subsidence of infectious diseases, than the history of typhus in our large English towns. At the same time, it must not be forgotten that there is much reason for believing that typhus fever is far more common even in London at the present time than is shown by the returns. Liverpool is a town in which this disease has never died out; and if we turn to Dr. Hope's most interesting Annual Report on the Health of Liverpool, and compare the case mortality of typhus fever there, where it is sufficiently common to keep people always on the watch for it, with the fatality in London, where it is so rare as to have almost slipped out of mind, we find that while in Liverpool only 11·8 of those attacked died, a third of the whole number of those notified in London succumbed to the disease. The conclusion is obvious, that there is a lot of mild typhus in London that is never recognised, and goes through its whole course unnotified and uncontrolled unless a localised outbreak happens to attract attention. It must be confessed that the difficulties in the way of diagnosis are considerable. In children the early symptoms are mild; and in adults, unless the temperature be taken, they closely resemble the effects of drink, while the disease is specially apt to occur among people of very dirty habits, in whom the rash may not be easy to identify. Often, also, by the time the case assumes any gravity of aspect, pneumonia or other complications have arisen, more or less marking the nature of the original disease. The possibility of obscure febrile ailments being really instances of typhus fever is, however, a matter to which the attention of practitioners in London may be usefully directed.

---

Dr. Hope, medical officer of health at Liverpool, has been made Professor of Public Health in connection with University College. The post is newly created. At Glasgow, Dr. Ralph Stockton has been appointed Professor of Materia Medica. Dr. Stockton has acted as assistant to Professor Fraser, of Edinburgh, and has had considerable experience in teaching his subject, to which research he has already materially added.

---

The preliminary programme of the sixteenth congress of the Sanitary Institute, to be held in Leeds, from September 14th to 18th, has now been issued. The president of the congress is Robert Farquharson, M.D. Edin., LL.D. Aberd., F.R.C.P. Lond., M.P., D.L., J.P. The congress will include three general addresses and lectures. The three sections meeting for two days each deal with "Sanitary Science and Preventive Medicine," "Chemistry," and other subjects. Six special conferences on "River Pollution," "Municipal Representatives," "Medical Officers of Health," "Municipal and County Engineers," "Sanitary Inspectors," and "Domestic Hygiene," will also be held. In connection with the congress an exhibition of apparatus and appliances relating to health and domestic use will be held.

